# Functional Translation of Exercise Responses from Exercise Testing to Exercise Training: The Test of a Model

**DOI:** 10.3390/jfmk6030066

**Published:** 2021-07-28

**Authors:** Tristan Tyrrell, Jessica Pavlock, Susan Bramwell, Cristina Cortis, Scott T. Doberstein, Andrea Fusco, John P. Porcari, Carl Foster

**Affiliations:** 1 Department of Exercise and Sport Science, University of Wisconsin-La Crosse, La Crosse, WI 54601, USA; tyrrell2697@uwlax.edu (T.T.); pavlock0615@uwlax.edu (J.P.); sbramwell@uwlax.edu (S.B.); sdoberstein@uwlax.edu (S.T.D.); jporcari007@gmail.com (J.P.P.); 2 Department of Human Sciences, Society and Health, University of Cassino and Lazio Meridionale, 03043 Cassino, Italy; c.cortis@unicas.it (C.C.); andrea.fusco@unicas.it (A.F.)

**Keywords:** exercise prescription, target heart rate, RPE, METs

## Abstract

Exercise prescription based on exercise test results is complicated by the need to downregulate the absolute training intensity to account for cardiovascular drift in order to achieve a desired internal training load. We tested a recently developed generalized model to perform this downregulation using metabolic equivalents (METs) during exercise testing and training. A total of 20 healthy volunteers performed an exercise test to define the METs at 60, 70, and 80% of the heart rate (HR) reserve and then performed randomly ordered 30 min training bouts at absolute intensities predicted by the model to achieve these levels of training intensity. The training HR at 60 and 70% HR reserve, but not 80%, was significantly less than predicted from the exercise test, although the differences were small. None of the ratings of perceived exertion (RPE) values during training were significantly different than predicted. There was a strong overall correlation between predicted and observed HR (r = 0.88) and RPE (r = 0.52), with 92% of HR values within ±10 bpm and 74% of RPE values within ±1 au. We conclude that the generalized functional translation model is generally adequate to allow the generation of early absolute training loads that lead to desired internal training loads.

## 1. Introduction

Exercise is a lifestyle factor that is beneficial to health [1,2,3,4]. It is beneficial on a dose–response basis, up to amounts of training of several times professional society guidelines [1,2] and only associated with health problems at levels consistent with heavy athletic training in middle-aged and older individuals [1,2,5]. In patients with chronic diseases, exercise forms a cornerstone of the treatment scheme of rehabilitation programs being of value both in terms of accelerating the rate of recovery [6], secondary prevention [7,8,9], and is even of value after contemporary medical care (percutaneous interventions and statins) is accounted for [10]. However, exercise training can present a significant risk of clinical events [11], particularly in the setting of unaccustomed heavy exercise in sedentary individuals [12] or in the setting of myocardial ischemia during exercise training [13]. Further, as with other behavioral interventions, new exercise programs have a disappointing pattern of compliance [14]. Although high-intensity exercise has recently become popular [15] and has well-documented efficacy [16], even in clinical populations [17,18,19], it is associated with at least a potentially increased risk of complications and reductions in program enjoyment and compliance [20].

In the setting of an increased risk based on age, risk factors, history of disease or symptoms, professional societies recommend graded exercise testing before beginning exercise programs, both to rule out clinically occult disease and to guide the exercise prescription [21]. The dominant model for subsequent exercise prescription is based on a percentage of the maximal exercise capacity (metabolic equivalents (METs)), heart rate (HR) or HR reserve (%HRR) [21], rating of perceived exertion (RPE) [22], or ventilatory threshold (VT) [23]. Recently, the talk test, which can be taken as a surrogate of VT, has been effectively used to prescribe exercise training [24,25] and to avoid ischemia during training [26].

Unfortunately, the same exercise workload during graded exercise testing (GXT) that produces a given HR, RPE, VT, or talk test response during incremental exercise, does not produce an equivalent response during sustained exercise training. The effect of cardiovascular drift dictates that the response to sustained exercise is often significantly larger than during brief stages at that same workload during a GXT. Potentially, this leads to training sessions that are harder than desired [27], which may, in turn, have adverse effects on safety and compliance. Accordingly, the exercise training workload must be reduced, downregulated, or “translated” in order to achieve desirable results during exercise training. Previous work from our laboratory has demonstrated a solution for translating exercise responses from GXT to ambulation and cycle ergometry [27,28], to arm–leg ergometry [29], to recreational activities [30]. A recent report from our laboratory demonstrated a potentially viable strategy to generalize the process of translating exercise test responses based on computing the MET cost during GXT and during training [31]. If this generalized model were shown to be accurate, then the process of translating either maximal or submaximal GXT responses to workloads useable on the first day of training would be more effective and safer. Accordingly, the intent of this study was to provide a systematic test of this generalized model on the basis of both %HRR and RPE responses.

## 2. Materials and Methods

The subjects for this study were 20 healthy, young adult volunteers. Although all were physically active, none were systematically trained athletes. The study protocol was approved by the Institutional Review Board for the Protection of Human Subjects at the University of Wisconsin-La Crosse (protocol # 45CFR46, approved 13 April 2021). All subjects provided written informed consent prior to participation. Characteristics of the subjects are presented in Table 1. The overall experimental approach was to start with MaxMETs, which is a normally measured variable during exercise testing, and then apply our predictive model for converting MaxMETs into the workload for exercise training [31] to test the degree to which exercise training responses (HR and RPE) during training fell within the desired range.

Each subject performed a GXT, to volitional fatigue, using a modified Bruce treadmill protocol [32]. During the test, HR and RPE (6–20 scale) [33] were measured at the end of each 1 min stage and blood pressure was measured every 3 min. During the test, respiratory metabolism was measured using open-circuit spirometry using a mixing chamber-based metabolic system (AEI Technologies, Pittsburgh, PA, USA). Integration of gas exchange values was performed every 30 s and VO_2_max was accepted as the highest 30 s value obtained during the test. The VT was computed using both the v-slope and ventilatory equivalent methods [34]. The HR reserve (HRR) was computed based on the observed maximal HR at the end of the test, and resting HR obtained in the standing position just before the test. The individual exercise time vs. HR curves were examined to determine the moment that the HR achieved reference values of 60% HRR, 70% HRR, and 80%HRR, which are surrogates for easy, moderate, and hard exercise. The RPE at these moments was also noted. The speed and grade of the treadmill belt at these moments were used to calculate the MET requirement based on conventional equations [21] and are referred to as GXT METs. The training workload was computed from the generalized functional translation model [31] as 72% of the GXT METs at each of the % HRR targets. The speed and grade required to achieve this MET level were computed by backward solving of the same equations [21]. From the solution, treadmill speeds between 1.79 and 2.23 m·s^−1^ (4–5 mph, 6.4–8 kmh) were avoided since solely walking or solely running is inconvenient in this speed range.

Each subject then performed three, randomly ordered 30 min training bouts (5 min warm-up at 1.33 m·s^−1^, 0% grade, 20 min at the targeted workload, and 5 min cool-down at 1.33 m^.−1^). HR and RPE were measured at 5 min intervals, and the HR and RPE at 15 min, 20 min, and 25 min were averaged and accepted as the HR and RPE response to the training workloads predicted by the generalized functional translation model.

The HR and RPE achieved during steady-state training at the intended easy, moderate, and hard workloads were compared to that calculated from responses during the GXT using repeated measures Analysis of variance (ANOVA). A *p*-value of <0.05 was accepted as statistically significant. When justified by ANOVA, pairwise comparisons were made using Tukey’s test. Correlations are calculated using the Pearson product–moment correlation.

## 3. Results

The predicted and achieved HR and RPE during the last 15 min of the easy, moderate, and hard training bouts are presented in Table 2. There were small but significant differences between predicted and achieved HR at the 60% HRR (easy) and 70%HRR (moderate) levels of intensity. There was no significant difference between predicted and achieved HR at the 80%HRR intensity. There were no significant differences between predicted and achieved RPE at any of the intensity levels.

**Figure 1:**
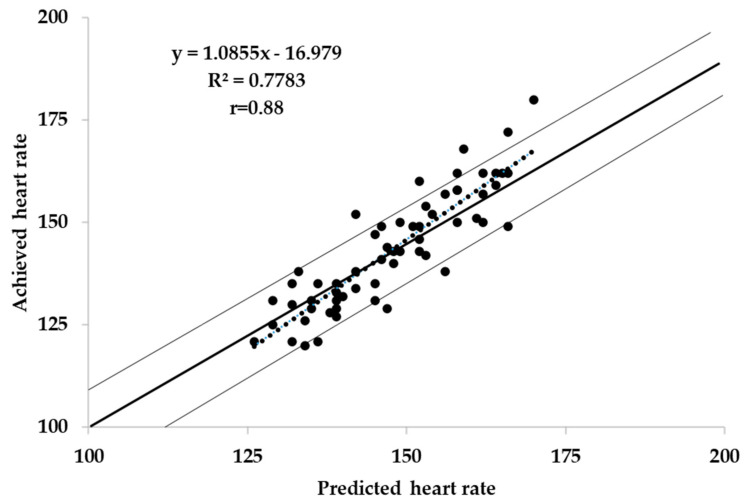
Predicted vs. achieved heart rate during the three intensities bouts.

A scatterplot of predicted vs. achieved HR for the combined results of the three intensities bouts is presented in Figure 1. There did not appear to be a significant bias in the pattern of responses, and the correlations between predicted and observed HR responses for easy (r = 0.52), moderate (r = 0.74), hard (r = 0.64), and combined (r = 0.88) were uniformly strong. Overall, 92% of observed HR values were within ±10 bpm of the predicted values.

**Figure 2:**
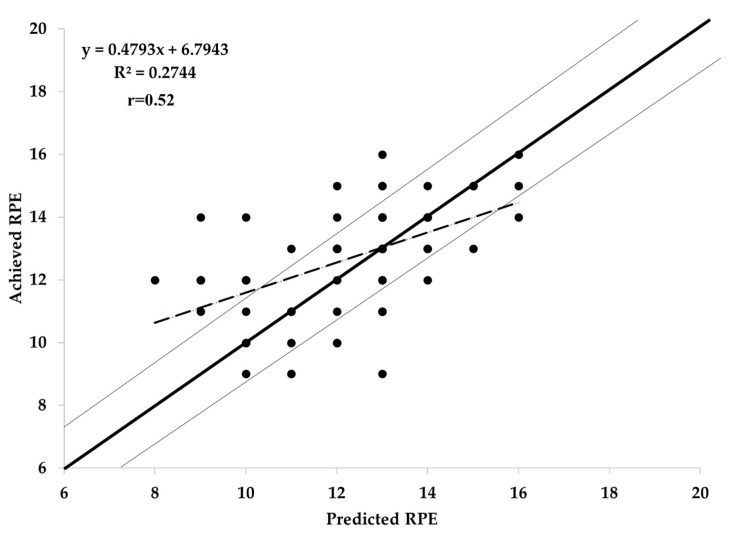
Predicted vs. achieved rating of perceived exertion (RPE) during the three intensities bouts.

A scatterplot of predicted vs. achieved RPE for the combined results of the three intensities bouts is presented in Figure 2. There did not appear to be a significant bias in the pattern of responses, and the correlations between predicted and observed RPE responses for easy (r = 0.66), moderate (r = 0.81), hard (r = 0.61), and combined (r = 0.88) were uniformly strong. Overall, 74% of the observations were within the ±1 RPE unit of the predicted values.

## 4. Discussion

The main finding of this study was that the generalized model for translating GXT responses into training workloads [31] appears to be accurate over the range of training intensities commonly prescribed in fitness and rehabilitation programs. While there was a bias for the predicted HR at the 60% (135 vs. 131) and 70% (148 vs. 143) HRR workloads to be slightly higher than observed, 92% of observed values were within ±10 bpm. Similarly, 74% of RPE values were within the ±1 RPE unit. Thus, the results support our earlier findings with more specific exercise translation approaches [27,28,29,30] but using a generalized approach.

While there was a small but significant overprediction of HR responses at the easy and moderate training loads, the error was small, and in a direction that would be acceptable clinically. One of the biggest concerns during exercise prescription is that workloads during the beginning days and weeks of a training program will be too heavy. This may result in reduced enjoyment [20] and may bias toward an increased likelihood of untoward events [11,12,13]. Thus, the tendency for the “translated” training load to be a little easier than predicted is a very acceptable error.

The primary limitation to the current results is attributable to a limitation of subject selection by the COVID-19 pandemic. In an idealized test of the generalized model, we would have selected fully sedentary individuals, or even patients from a rehabilitation program, as these are the individuals who actively need a strategy for translating GXT results into training prescriptions. However, restrictions on laboratory use dictated that younger and more active students served as the subjects. However, as none of the subjects was systematically training for sports competition, and as their VO_2_max spanned a considerable range of fitness, we feel that the experimental test of the generalized functional translation model remains valid.

The generalized functional translation model tested in this study was based on the results of a maximal GXT, as had been the results of the progenitor studies [27,28,29,30]. Contemporary practice in both fitness and rehabilitation communities is not to have maximal GXT results available. Where a preliminary GXT is performed, it is often submaximal in nature, limited to an RPE of 15 (hard). However, the strong relationship between the progression of RPE and relative exercise intensity [35,36] and the strong relationship between the talk test and relative exercise intensity [24,25,36] suggest that picking target values for RPE or the talk test from a submaximal GXT and then applying the generalized functional translation model is likely to yield the same prescriptive result as a maximal effort GXT [25].

## 5. Conclusions

The results of this study suggest that a generalized model for translating GXT results to exercise training loads, based on calculated MET values [31], yields both HR and RPE responses during training bouts that are close to predicted values. Therefore, the results suggest a prescriptive strategy based on 70–75% of the MET requirement at that level of internal training load during a GXT is likely to make the beginning portions of an exercise training program both more pleasant and safer while still being effective.

## 6. Practical Applications

As an example of the use of the functional translation model, which is validated in the present data, consider the following: A sedentary, although healthy, person performs a GXT (without handrail support) using a Balke type treadmill protocol (a constant speed with grade increments every 2 min) with the results in Table 3.

The target HR (THR) is calculated at 70% HRR as follows:

THR = [(160 − 70) × 0.7] + 70

THR = [90 × 0.7] + 70 = 133 bpm

METs are calculated per standard equations [21] as

VO_2_ = [(speed (m·min^−1^) × 0.1] + [speed (m·min^−1^) × 1.8 × grade/100] + 3.5

For example, at 4% grade (e.g., 70%HRR),

VO_2_ = [80.4 × 0.1] + [80.4 × 1.8 × 0.04] + 3.5

VO_2_ = 8.4 + 5.8 + 3.5 = 17.7 mL·min^−1·^kg^−1^ = 5.1 METs

Applying the generalized functional translation model, the training intensity becomes

5.1 × 0.72 = 3.7 METs = 13.0 mL·min^−1·^kg^−1^

Solving for grade at a walking speed of 1.34 m·s^−1^,

13.0 = [80.4 × 0.1] + [80.4 × 1.8 × grade] + 3.5

13.0 = 8.0 + [144.7 × grade] + 3.5

(13.0 − 8.0 − 3.5)/144.7 = grade

1.5/144.7 = grade = 0.01 = 1%

Thus, with the tabled GXT results, one would expect ~70% HRR with an RPE of 11–12 at a walking speed of 1.34 m·s^−1^ (3.0 mph or 4.8 kmh) at a grade of 1%. While this may not provide the specific results desired, it does provide a reasonable candidate for how to prescribe the first workouts in the training facility.

## Figures and Tables

**Table 1:** Mean and standard deviation of the characteristics of the subjects.

Characteristics	Males (*n* = 10)	Females (*n* = 10)
Age (years)	23.5 ± 2.2	22.3 ± 0.9
Height (cm)	183.1 ± 7.3	167.6 ± 6.7
Weight (kg)	82.3 ± 18.4	70.0 ± 13.2
VO_2_max (ml·kg^−1^·min^−1^)	53.3 ± 6.1	40.3 ± 5.3
HRmax (bpm)	189 ± 7	185 ± 5
RPEmax	19.9 ± 0.2	19.9 ± 0.2

VO_2_max: maximal oxygen uptake; HRmax: maximal heart rate; RPEmax: maximal rating of perceived exertion.

**Table 2:** Mean and standard deviation of the comparison between the predicted and achieved values of heart rate (HR) and rate of perceived exertion (RPE) at the 60%, 70%, and 80% of the heart rate reserve (% HRR) intensities.

% HRR	Predicted HR	Achieved HR	Predicted RPE	Achieved RPE
60%	135.1 ± 4.3	130.2 ± 7.6 *	11.8 ± 1.2	11.2 ± 1.5
70%	147.9 ± 4.6	142.8 ± 8.9 *	13.0 ± 1.4	12.4 ± 1.7
80%	160.4 ± 5.4	157.3 ± 9.7	14.1 ± 1.1	13.8 ± 1.3

* = significantly (*p* < 0.05) different from predicted HR.

**Table 3:** Example of a subject’s speed, grade, metabolic equivalents (METs), heart rate (HR), and rating of perceived exertion (RPE) response values during a graded exercise testing using a Balke type treadmill protocol.

Speed (m·s^−1^)	Grade (%)	METs [21]	HR	RPE
0	0	1	70	6
1.34	0	3.3	95	8
1.34	2	4.1	115	10
1.34	4	4.9	133	12
1.34	6	5.8	150	14
1.34	8	6.6	155	16
1.34	10	7.4	160	18.5

## Data Availability

The data presented in this study are available on request from the corresponding author.
